# Research article proteomics-based plasma biomarkers for predicting CRKP infection in ICU sepsis patients

**DOI:** 10.3389/fphar.2026.1786611

**Published:** 2026-03-13

**Authors:** Zhongan Mao, Kai Yao, Lei Wang, Yujie Wang, Yongfang Yuan

**Affiliations:** 1 Department of Pharmacy, Shanghai 9th People’s Hospital, Shanghai Jiao Tong University School of Medicine, Shanghai, China; 2 College of Clinical Pharmacy, Shanghai Jiao Tong University School of Medicine, Shanghai, China

**Keywords:** CRKP, ICU, MDRO, proteomics, sepsis

## Abstract

**Background:**

Early differentiation between carbapenem-resistant *Klebsiella pneumoniae* (CRKP) and carbapenem-sensitive *K. pneumoniae* (CSKP) infections is critical due to limited treatment options and high mortality associated with CRKP. Current diagnostic methods are slow and insufficient for timely clinical decision-making, especially in ICU settings. Identifying reliable biomarkers for rapid discrimination is urgently needed.

**Methods:**

We performed plasma proteomic profiling of ICU sepsis patients infected with CRKP or CSKP using data-independent acquisition (DIA) mass spectrometry. Significantly differentially expressed proteins (DEPs) underwent Gene Ontology (GO), Kyoto Encyclopedia of Genes and Genomes (KEGG), and Disease Ontology (DO) functional annotation and enrichment analyses. Hub proteins were identified through protein-protein interaction network analysis. Protein biomarkers for constructing a diagnostic model by logistic regression analysis were further selected by XGboost and Lasso. The model was then evaluated for discrimination, calibration, and clinical utility by area under curve (AUC), the Hosmer–Lemeshow goodness-of-fit test and calibration curve, and decision curve, respectively.

**Results:**

A total of 1,432 proteins and 13,482 peptides were identified in the plasma samples. Among these, 28 DEPs were detected, including 16 upregulated and 12 downregulated proteins. Functional enrichment analysis indicated that these DEPs were primarily associated with neural and cardiovascular pathways. Using a combination of XGBoost and LASSO algorithms, 10 protein biomarkers were selected to construct a diagnostic model. The proteins of the optimal diagnostic model included PLXNB1 and S100A1. Notably, a simplified two-protein model demonstrated excellent diagnostic accuracy with an AUC exceeding 0.90 in both training and testing cohorts. The Hosmer–Lemeshow goodness-of-fit test yielded p-values of 0.825 and 0.295 in the training and testing sets, respectively, indicating good model calibration.

**Conclusion:**

PLXNB1 and S100A1 serve as promising plasma biomarkers for early, non-culture-based differentiation of CRKP and CSKP infections. Their integration into clinical workflows could improve rapid diagnosis and guide targeted therapy in critically ill sepsis patients.

## 1 Introduction

*Klebsiella pneumoniae* (KP) is a major opportunistic pathogen commonly associated with nosocomial infections such as pneumonia, urinary tract infections, bloodstream infections, and sepsis (Diekema et al., 2019). Although carbapenems, a class of broad-spectrum antibiotics, are the recommended first-line treatment for severe KP infections, their widespread use has driven the global emergence of carbapenem-resistant *Klebsiella pneumoniae* (CRKP), posing a significant threat to antimicrobial efficacy and public health. The global rise (Li et al., 2022) of CRKP—with detection rates in China increasing from 9.0% in 2011 to 23.4% in 2024 (http://www.chinets.com/Data/GermYear)—is concerning, as CRKP infections are associated with significantly higher mortality rates compared to those caused by carbapenem-susceptible *Klebsiella pneumoniae* (CSKP) (Xu et al., 2017). Critically ill patients in the intensive care unit (ICU) are particularly susceptible to CRKP infections due to impaired immunity, extensive antibiotic use, and invasive procedures such as tracheal intubation and tracheotomy (Mangioni et al., 2023; Liao et al., 2023). Such infections may eventually lead to sepsis, further exacerbating disease progression and contributing to high mortality and increased healthcare costs, especially given the limited therapeutic options. (Xu et al., 2017; Kong et al., 2025). Therefore, early prediction of CRKP infection in ICU sepsis patients is crucial for timely and accurate diagnosis, and a reliable model with high predictive accuracy may help reduce infection risk and improve clinical management in critically ill patients.

In recent years, the development of prediction models for early identification, monitoring, and prognosis has become a research hotspot in the field of CRKP infection prevention and control (Liao et al., 2023; Chen et al., 2022; Guan et al., 2025). While some studies have explored simple inflammatory indices derived from complete blood counts as accessible tools for predicting disease severity in various acute conditions (The predictive value of inflammatory, 2023; Vural et al., 2024), these markers often lack the molecular specificity required to distinguish between specific bacterial resistance phenotypes. Furthermore, most reported models rely on traditional logistic regression analyses, which may struggle to capture complex, non-linear biological relationships. Consequently, there remains a lack of clinically validated biomarkers and advanced analytical frameworks for early risk assessment of CRKP infection in ICU patients.

To address these limitations, proteomics has emerged as a superior alternative. With its advantages of high throughput, precise quantification, and molecular-level resolution, proteomics provides essential insights into complex diseases by uncovering potential biomarkers, thereby offering new perspectives for disease diagnosis and the elucidation of pathological mechanisms (Wei et al., 2023; Zhang et al., 2023; Shu et al., 2020). Moreover, as interest grows in understanding antimicrobial resistance, the integration of proteomic sequencing with machine learning is increasingly being used. This approach helps identify subtle protein differences between drug-sensitive and resistant *Klebsiella pneumoniae* strains that traditional clinical variables might overlook, offering insights into resistance mechanisms and potential treatment targets (Hao et al., 2022; Wang et al., 2025).

However, proteomic studies specifically focusing on the early prediction of CRKP infection in ICU sepsis patients still require further investigation. Given the current lack of accurate biomarkers for CRKP infections in ICU patients, there is an urgent need for novel methods to identify high-risk individuals. Leveraging proteomics’ ability to reveal disease-associated molecular features, we conducted a proteomics-based study to predict CRKP infection risk in ICU patients. Using data-independent acquisition (DIA) proteomics, we identified differentially abundant plasma proteins in sepsis patients infected with CRKP and developed a proteomic model to distinguish them from patients infected with CSKP. These predictive biomarkers provide more objective and quantitative criteria for assessing CRKP infection risk and offer insights into underlying pathophysiological mechanisms.

## 2 Materials and methods

### 2.1 Patient information and sample collection

This was a multicenter retrospective observational study conducted at two tertiary hospitals in China: the ICU of Ruijin Hospital, Shanghai Jiao Tong University School of Medicine, and the ICU of The Ninth People’s Hospital Affiliated to Shanghai Jiao Tong University School of Medicine. The study period spanned from October 2024 to June 2025. The study protocol was approved by the Ethics Committee of Ruijin Hospital (Approval No.: 2024372) and the Ethics Committee of The Ninth People’s Hospital (Approval No.: SH9H-2025-T360-2). All procedures were carried out in accordance with the Declaration of Helsinki (2013 revision). Patients were eligible for inclusion if they (1) were aged over 18 years and (2) had sepsis caused by *Klebsiella pneumoniae*. Exclusion criteria included (1) isolation of more than one pathogen during the ICU stay, and (2) pregnancy or lactation. Eligible patients were classified into two groups: the CRKP group and the CSKP (control) group. CRKP was defined as *K. pneumoniae* strains resistant to at least one carbapenem antibiotic, including ertapenem, meropenem, or imipenem (Bassetti et al., 2016).

A total of 18 patients were initially included in the CSKP (control) group and 14 in the CRKP group. To control for confounding variables, propensity score matching was performed using IBM SPSS Statistics (version 27), with age and gender as matching factors and a caliper value of 0.2. After one-to-one matching, 14 matched pairs (14 CRKP vs. 14 CSKP) were obtained. Plasma samples from the 14 CRKP-infected and 14 CSKP-infected patients were collected for mass spectrometry and bioinformatics analysis. Protein biomarkers were screened using XGBoost and LASSO algorithms to construct a diagnostic model, and the model’s performance was subsequently evaluated.

### 2.2 Sample preparation

Protein enrichment was performed using Superparamagnetic Iron Oxide Nanoparticles (SPIONs), which based on the “protein corona” effect. Unlike standard immunodepletion methods (which specifically remove high-abundance proteins like albumin and IgG but are costly and may non-specifically remove bound low-abundance proteins), SPIONs provide a high-throughput, cost-effective strategy to compress the dynamic range of plasma proteins. The unique surface physicochemical properties of these nanoparticles allow them to preferentially adsorb low-abundance proteins (the “deep proteome”) while excluding the vast majority of high-abundance background proteins. Briefly, 20 μL of plasma sample was diluted in loading buffer (10 mM Tris-Cl, 1 mM EDTA, 150 mM KCl, 0.05% CHAPS) and incubated with 1 mg of magnetic beads at 37 °C for 1 h. After incubation, the beads were washed twice with the same loading buffer and once with CHAPS-free buffer (10 mM Tris-Cl, 1 mM EDTA, 150 mM KCl). The magnetic beads were then captured using a magnetic rack, and the supernatant was discarded to retain the protein-bound beads. For protein reduction and alkylation, lysis buffer (1% sodium deoxycholate (SDC), 100 mM Tris-HCl, pH 8.5, 10 mM tris(2-carboxyethyl)phosphine (TCEP), 40 mM 2-chloroacetamide (CAA) was added, followed by incubation at 60 °C for 30 min. The sample was then diluted with an equal volume of (ddH_2_O) to reduce the SDC concentration below 0.5%, and 1 μg of trypsin (SignalChem, Richmond, BC, Canada) was added for enzymatic digestion at 37 °C overnight. On the following day, the digestion was terminated by adjusting the pH to 6.0 using trifluoroacetic acid (TFA, Sigma-Aldrich, St. Louis, MO, United States). After centrifugation, the supernatant containing peptides was desalted using a self-made styrene divinylbenzene-reverse phase sulfonate (SDB-RPS) column. The eluted peptides were vacuum-dried and stored at −20 °C for subsequent analysis.

### 2.3 Liquid chromatography-Tandem mass spectrometry (LC-MS/MS) analysis

All samples were analyzed using a timsTOF Pro mass spectrometer (Bruker Daltonics, Bremen, Germany), a hybrid trapped ion mobility spectrometry (TIMS) quadrupole time-of-flight instrument, coupled with an UltiMate 3,000 RSLCnano system (Thermo Fisher Scientific, Waltham, MA, United States) and a CaptiveSpray nano ion source (Bruker Daltonics, Bremen, Germany). Peptides were first loaded onto a C18 trap column (75 μm × 2 cm, 3 μm particle size, 100 Å pore size; Thermo Fisher Scientific, Waltham, MA, United States) and subsequently separated on a reversed-phase C18 analytical column (75 μm × 15 cm, 1.7 μm particle size, 100 Å pore size; IonOpticks, Fitzroy, VIC, Australia). Chromatographic separation was performed at a flow rate of 300 nL/min using a binary solvent system: mobile phase A consisted of 0.1% formic acid in water, and mobile phase B was 0.1% formic acid in acetonitrile (ACN). The mass spectrometer was operated in data-independent acquisition parallel accumulation-serial fragmentation (diaPASEF) mode. The capillary voltage was set to 1500 V, and MS and MS/MS spectra were acquired over an m/z range of 100–1700. The ion mobility range was set from 0.6 to 1.6 Vs/cm^2^, with both the accumulation and ramp times configured at 50 ms. The diaPASEF acquisition method was defined using timsControl software (Bruker Daltonics, Bremen, Germany) in the m/z–ion mobility dimension. Collision energy was ramped linearly based on ion mobility, from 59 eV at 1/K_0_ = 1.6 Vs/cm^2^ to 20 eV at 1/K_0_ = 0.6 Vs/cm^2^.

### 2.4 Library search

DIA raw files were analyzed using DIA-NN (version 1.9.2) in library-free mode. Spectral searches were performed against the HUMAN protein sequence database downloaded from Uniprot (20230619). The search parameters were largely set to default, with the following specific configurations:

1. In silico spectral library generation was enabled for precursor ion prediction.
2. Enzyme specificity was set to Trypsin/P, allowing up to 2 missed cleavages.
3. Fixed modification: Carbamidomethyl on C.
4. Variable modifications: Oxidaton on M and protein N-terminal acetylation.
5. Mass accuracy for MS1 and MS2 was set to 15 ppm.
6. Match-between-runs (MBR) and heuristic protein inference were enabled to improve identification consistency across samples.
7. Precursor-level FDR was controlled at 1% to ensure high-confidence identifications.

Protein intensities were normalized using the MaxLFQ algorithm to enable accurate label-free quantification.

### 2.5 Data preprocessing

The quantitative protein matrix was log-transformed following the removal of contaminant proteins. Missing values were then imputed using a left-censored approach, simulating a normal distribution near the detection limit of the mass spectrometer. Specifically, the mean and standard deviation of observed intensities were calculated, and a new distribution was generated with a downshift of 1.8 standard deviations and a width of 0.25 standard deviations. Proteins with more than 50% missing values in both groups were excluded prior to imputation to ensure data robustness. The resulting matrix was used for subsequent statistical analyses.

### 2.6 Functional annotation and enrichment analysis

Functional annotation and enrichment analysis of differentially expressed proteins (DEPs) were performed to explore their associated biological functions and molecular mechanisms. Significantly enriched functions and pathways were identified using the hypergeometric test with a threshold of P < 0.05. Functional annotation was conducted using the following databases:

1. Gene Ontology (GO, http://www.geneontology.org/): This knowledgebase provides hierarchical classification of gene functions in three categories: Biological Process (BP): biological objectives or processes in which gene products are involved; Cellular Component (CC): subcellular structures, locations, or macromolecular complexes; and Molecular Function (MF): molecular-level activities of gene products.
2. Kyoto Encyclopedia of Genes and Genomes (KEGG, https://www.kegg.jp/kegg/): A comprehensive database describing molecular-level biological systems, including pathways related to metabolism, genetic information processing, environmental responses, cellular processes, human diseases, and drug development.
3. Disease Ontology (DO, https://disease-ontology.org/): A knowledgebase used to annotate gene involvement in human diseases.

Protein–protein interaction (PPI) networks of DEPs were analyzed using the STRING database, which integrates interaction evidence from experimental data from multiple public databases, including text mining data, neighborhood gene, co-expression gene and other protein-related database data. The PPI networks were visualized using Cytoscape software. The CytoHubba plugin was applied to identify key hub proteins using four algorithms: Degree, DMNC, MCC, and MNC. Proteins identified by at least three of these algorithms were considered hub proteins.

### 2.7 Statistical analysis and biomarker selection

Clinical variables were described as follows: continuous variables with normal distribution were presented as mean ± standard deviation, while those with non-normal distribution were expressed as median and interquartile range (IQR). Categorical variables were reported as percentages. To compare protein abundance between groups, Student’s t-test was applied when data were normally distributed in both groups; otherwise, the Wilcoxon rank-sum test was used.

Protein biomarker screening was conducted in three steps: 1) Differential expression analysis based on fold change (FC) and two-sided P < 0.05; 2) Extreme Gradient Boosting (XGBoost) was applied to further refine the candidate proteins; 3) Least Absolute Shrinkage and Selection Operator (LASSO) regression with cross-validation was used to select the final biomarker set for model construction.

The dataset was randomly split in a stratified manner by group into a training set (75%) and a testing set (25%). In the training set, multivariate logistic regression was used to construct diagnostic models based on different combinations of selected protein biomarkers. The performance of each model was evaluated using the area under the receiver operating characteristic curve (AUC) and the Brier score, both calculated via cross-validation. The Brier score quantifies the accuracy of probabilistic predictions by measuring the mean squared difference between predicted probabilities and actual outcomes, reflecting the model’s calibration performance. Finally, the model’s overall performance was assessed in both training and testing sets from three perspectives: receiver operating characteristic (ROC) curves, the Hosmer–Lemeshow goodness-of-fit test and calibration curves, and decision curve analysis (DCA). Formal subgroup analysis was not performed due to sample size limitations, which precludes a reliable assessment of model stability across strata. All statistical analyses were performed using R software (version 4.4.3) and Python (version 3.10.12).

## 3 Results

### 3.1 Differential protein analysis

**FIGURE 1:**
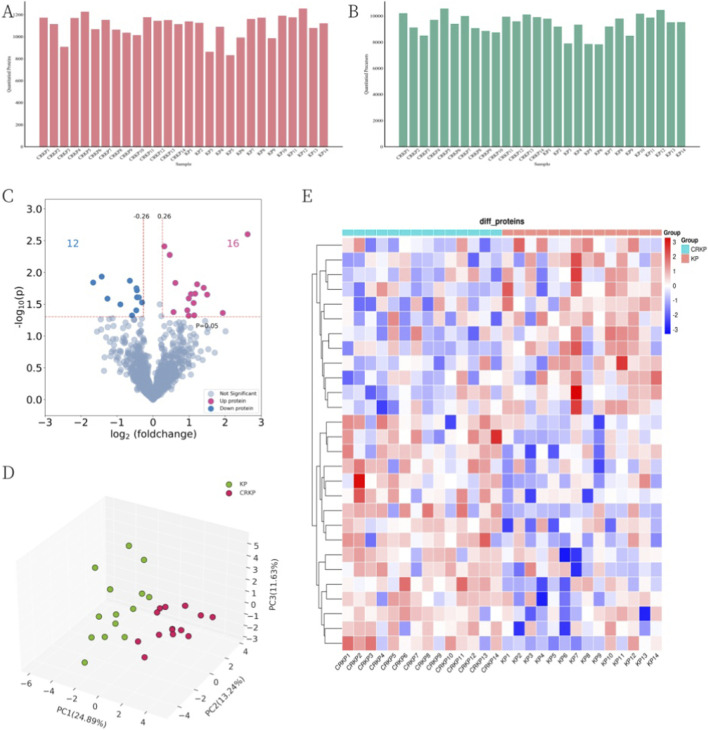
Differential analysis of proteins expression levels between disease and control group. **(A)** The number distribution of quantitative proteins. **(B)** The number distribution of quantitative peptides. **(C)** The volcano plot of DEPs. Each point represented a protein, where red points represented upregulated proteins and blue points represented downregulated proteins. The threshold values in the graph were |log2FC| ≥ 0.26 (vertical dashed line) and P < 0.05 (horizontal dashed line). **(D)** PCA plot of samples featured by DEPs. Each point represented one sample. **(E)** Quantitative heatmap of DEPs between samples. Rows represented DEPs, columns represented different samples, and colors indicated the expression levels of DEPs.

We profiled the serum proteome of all participants (n = 28) using a DIA strategy. In total, 1,432 proteins and 13,482 peptides were quantified across all samples (Figures 1A,B). After excluding proteins with >50% missing values in both groups, 1,202 proteins remained for subsequent analyses. Compared with the CSKP group, 28 proteins were identified as significantly differentially expressed in the CRKP group (|log_2_FC| ≥ 0.26, p < 0.05), including 16 upregulated and 12 downregulated proteins (Figure 1C).

Principal component analysis (PCA) based on these 28 DEPs demonstrated a clear separation between the CRKP and CSKP groups (Figure 1D). Although the variance explained by PC1 and PC2 is relatively low, this is typical for high-dimensional proteomics data with inherent biological heterogeneity. Crucially, the clear separation between groups demonstrates that these components capture the primary biological differences. We examined subsequent components (e.g., PC3, PC4) but found they primarily reflected individual variability rather than further distinguishing the study groups. Consistently, hierarchical clustering heatmap analysis revealed distinct expression patterns of DEPs between the two groups (Figure 1E).

### 3.2 Annotation and enrichment analysis

**FIGURE 2:**
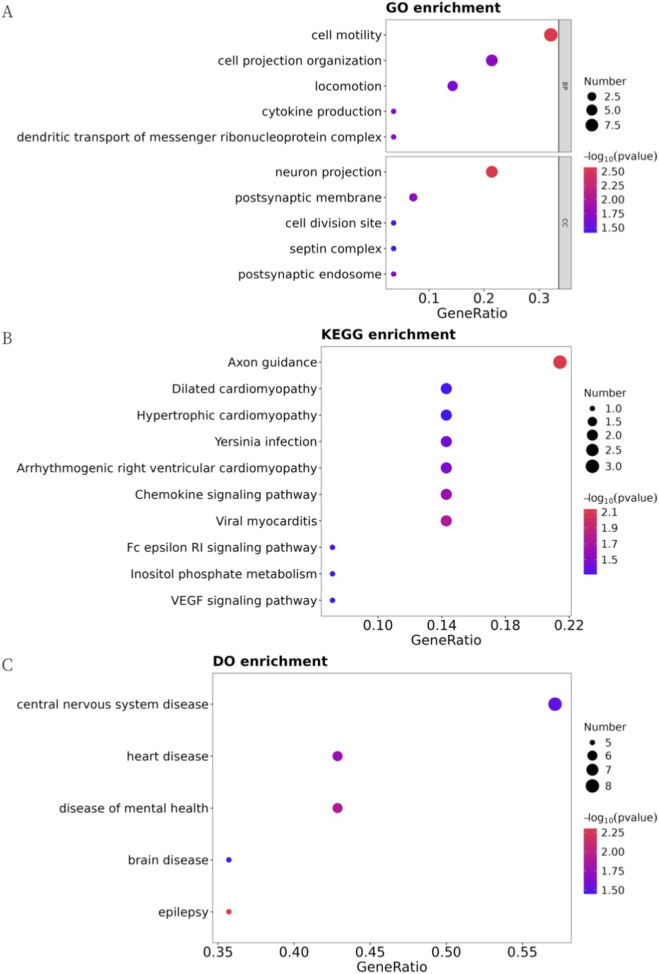
Partial results of enrichment analysis of DEPs (hypergeometric test P < 0.05). **(A)** GO-based enrichment analysis of DEPs(two-sided hypergeometric test; p < 0.05), GO terms were sorted by p-value, and top 5 terms of each category were displayed. **(B)** KEGG-based enrichment analysis of DEPs(two-sided hypergeometric test; p < 0.05), KEGG terms were sorted by p-value, and top 15 terms were displayed. **(C)** DO-based enrichment analysis of DEPs(two-sided hypergeometric test; p < 0.05), DO terms were sorted by p-value, and top 15 terms were displayed.

To explore the biological relevance of these DEPs, Gene Ontology (GO), Kyoto Encyclopedia of Genes and Genomes (KEGG), and Disease Ontology (DO) enrichment analyses were performed (P < 0.05, hypergeometric test). GO terms were mainly enriched in biological processes such as cell motility, cell projection organization, and locomotion, and in cellular components such as neuron projection, postsynaptic membrane, and septin complex, while no significant molecular function enrichment was detected (Figure 2A). KEGG pathway analysis revealed enrichment in axon guidance, hypertrophic cardiomyopathy, and dilated cardiomyopathy (Figure 2B). DO analysis indicated associations with central nervous system diseases, heart diseases, and mental health disorders (Figure 2C). These results suggest that although the DEPs are predominantly annotated in neural and cardiac processes, their roles in infection-related pathophysiology remain to be clarified.

### 3.3 Protein-protein interaction network analysis of DEPs

**FIGURE 3:**
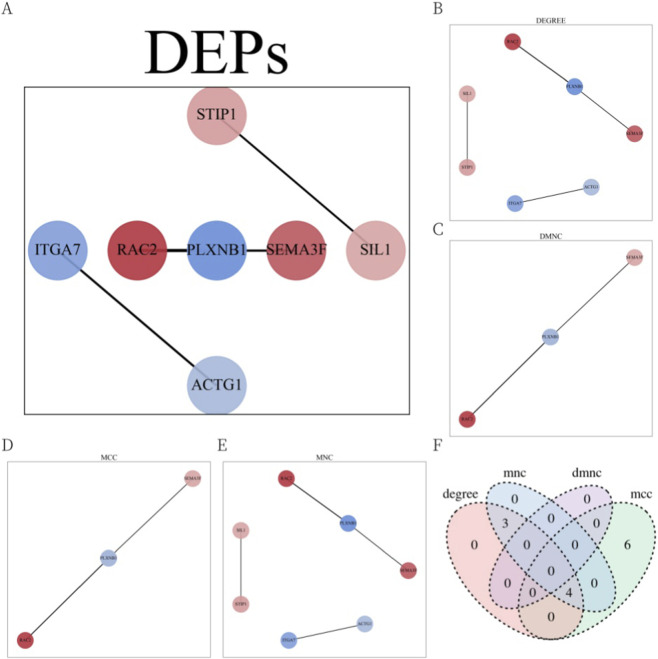
PPI networks analysis results of DEPs. **(A)** PPI networks of DEPs. Circles represented proteins, the connecting lines’ thickness represented the credibility of the evidence for PPI, and the color of the circles represented the upregulation (red)/downregulation (blue) trend of DEPs. **(B)** Key proteins from the PPI network selected by the DEGREE algorithm. **(C)** Key proteins from the PPI network selected by the DMNC algorithm. **(D)** Key proteins from the PPI network selected by the MCC algorithm. **(E)** Key proteins from the PPI network selected by the MNC algorithm. **(F)** Venn diagram of the key proteins from the four algorithms. Hub proteins selected by intersection of there and more algorithms.

To explore potential functional relationships among the DEPs, a PPI network was constructed using CytoScape software based on STRING database data (Figure 3A). Four CytoHubba algorithms—DEGREE, DMNC, MCC, and MNC—were applied to identify the top 10 key proteins in the network (Figures 3B–E). The intersection of these results, visualized via a Venn diagram (Figure 3F), revealed four hub proteins present in at least three algorithms: PLXNB1, RAC2, STIP1, and SEMA3F. These hub proteins may represent central nodes in CRKP-related host response pathways and merit further investigation.

### 3.4 Prediction of disease risk

**FIGURE 4:**
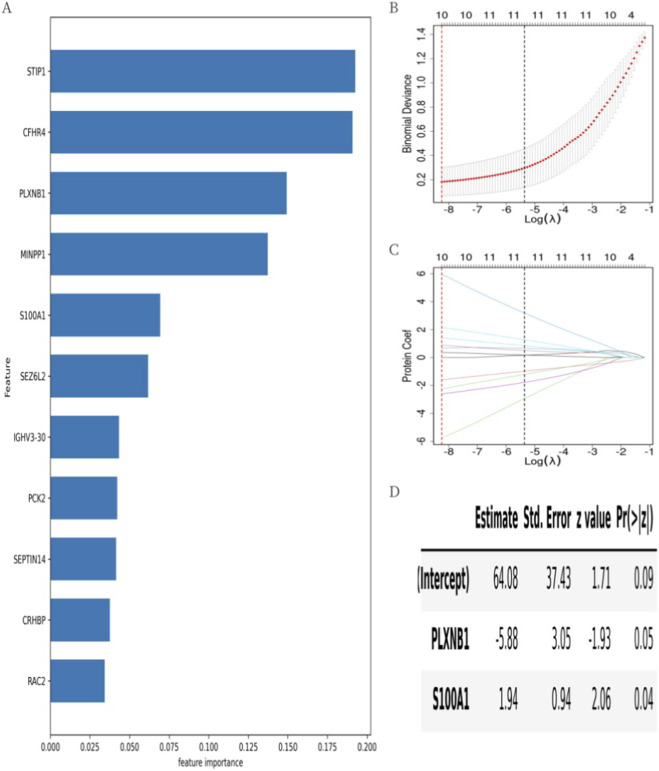
Predictive model construction process and model parameter. **(A)** Feature importance ranking of screened proteins with importance score >0 by XGBoost. The feature importance of the proteins gradually decreased from top to bottom. **(B)** The mean square error changed with Log(λ) during the screening of proteins by the Lasso regression. The upper horizontal axis was the number of screened proteins. The number of proteins at the minimum mean square error value was the number of proteins screened for subsequent analysis by Lasso regression. **(C)** The regression coefficients changed with Log(λ) during screening of proteins by Lasso regression. **(D)** The parameters of the optimal Logistic regression model. The “Estimate” was the regression coefficient, the “Std.Error” was the standard error of the regression coefficient, the “z value” was the test statistic of the Z-test for the regression coefficients, and the “Pr (>|z|)” was the P-value of the Z-test for the regression coefficients.

All samples were randomly divided into a training set and a testing set at a 3:1 ratio. In the training set, the XGBoost machine learning model identified 11 important proteins from the 28 DEPs, ranked by feature importance (Figure 4A). Next, Lasso regression with 5-fold cross-validation further reduced the number to 10 key proteins: CFHR4, PLXNB1, MINPP1, S100A1, SEZ6L2, IGHV3-30, PCK2, SEPTIN14, CRHBP, and RAC2 (Figures 4B,C).

Random combinations of these 10 proteins were used to construct logistic regression models. The optimal model was selected based on both the discrimination index (AUC) and the calibration index (Brier score), each calculated using three-fold cross-validation. Specifically, we prioritized the model that achieved the lowest Brier score among those with high AUC values to ensure the reliability of predicted probabilities. The final diagnostic model included PLXNB1 and S100A1 (Figure 4D).

### 3.5 Validation of diagnostic model

We evaluated the model’s discrimination, calibration, and clinical utility in both training and testing sets.

**FIGURE 5:**
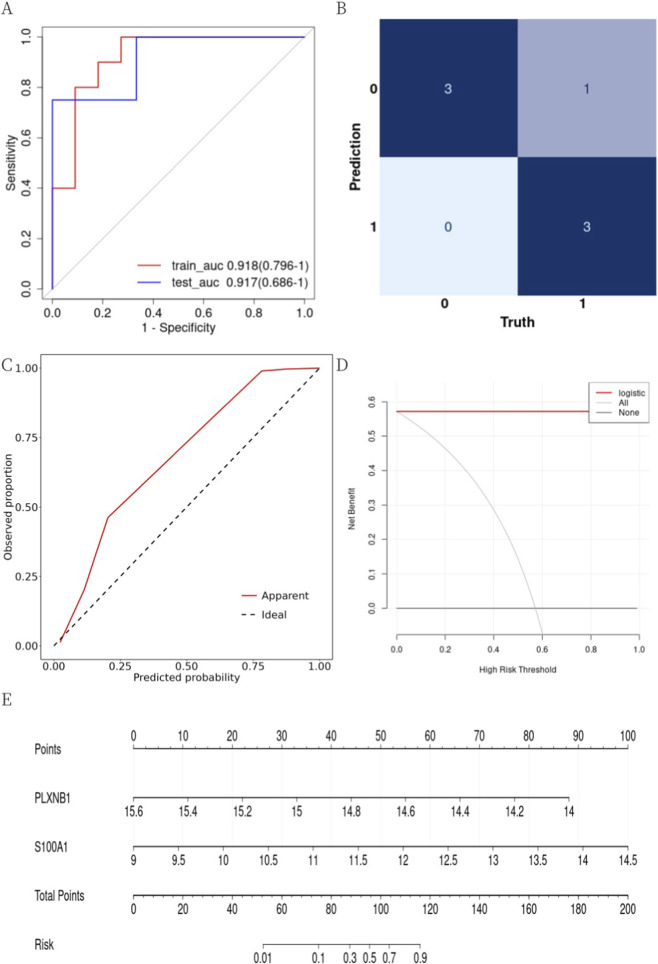
Evaluation of the diagnostic model. **(A)** ROC curves for the training and testing sets. The horizontal axis was the 1-specificity and the vertical axis was the sensitivity. **(B)** Confusion matrix of the prediction results in the testing set. The horizontal axis represented the actual classes and the vertical axis represented the predicted class. **(C)** Calibration curves of the diagnostic models in the test set. The dashed line was the ideal curve, the solid red line was the actual calibration curve of the model. **(D)** Decision curve of the diagnostic model in the test set. The horizontal axis was the threshold probability, the vertical axis was the benefit net, the black line represented 0 benefit net when no interventions were implemented for all, the grey line represented to the benefit net with a negative slope curve when interventions were implemented for all, and the red line represented the benefit net of the model. **(E)** The nomogram provided a visual display of the Logistic regression model. The “Points” represented scores corresponding with the values of predictor variables. The “Total Points” represented the sum scores of all predictor variables. The “Risk” represented the risk probability corresponding with the total scores.

ROC curves assessed discrimination. The AUCs were 0.918 (0.796–1) for the training set and 0.917 (0.686–1) for the testing set (Figure 5A), indicating excellent discrimination (AUC ≥0.90).

The confusion matrix showed an accuracy of 86% in both sets (Figure 5B). The Brier scores for the training and testing sets were 0.082 and 0.085, respectively, indicating a high degree of correspondence between predicted probabilities and actual outcomes. The Hosmer–Lemeshow goodness-of-fit test yielded p = 0.825 for the training set and p = 0.295 for the testing set. A p-value >0.05 indicates that there is no significant difference between predicted and observed outcomes, suggesting good model fit. Calibration curves (Figure 5C) were close to the 45° diagonal, indicating good agreement between predicted and observed outcomes. Decision curve analysis (DCA) assessed clinical utility. The red curve (model) was above the grey curve (intervention for all), suggesting higher net benefit when using the model to guide intervention (Figure 5D). Based on multivariate logistic regression, we developed a nomogram to predict event probability (Figure 5E). Each variable corresponded to a score, and the sum of scores indicated the estimated probability.

In summary, we identified a distinct proteomic profile differentiating CRKP-infected ICU patients from CSKP-infected patients, and developed a robust diagnostic model based on two key proteins—PLXNB1 and S100A1—with excellent predictive performance. These findings highlight potential biomarkers for the early identification of CRKP infection risk in ICU sepsis patients.

## 4 Discussion

Early differentiation between CRKP and CSKP infections is crucial due to limited therapeutic options and high mortality. Traditional body fluid cultures (Li et al., 2024) are time-consuming and inadequate for early screening, while alternative approaches like risk factor analysis or mNGS (Chen et al., 2022; Wu et al., 2022; Wan et al., 2024) are often costly or impractical in ICU settings. Although hemogram-derived markers have shown utility in other emergency settings (The predictive value of inflammatory, 2023; Vural et al., 2024), they lack the specificity to discriminate bacterial resistance patterns. In this study, we identified a distinctive plasma proteomic signature and developed a two-protein diagnostic model (PLXNB1 and S100A1) with excellent discrimination (AUC >0.90) and calibration in both training and testing.

Functional enrichment analysis revealed that the differentially expressed proteins (DEPs) were primarily associated with neural and cardiovascular pathways, reflecting well-known sepsis complications such as neurological dysfunction and septic cardiomyopathy (Sekino et al., 2022; Carbone et al., 2022; Wang et al., 2022). Sepsis-induced cerebral dysfunction involves blood–brain barrier (BBB) disruption, neuroinflammation, and the accumulation of amyloid β (Aβ) and tau proteins (Sekino et al., 2022). Similarly, septic cardiomyopathy stems from hyperinflammatory responses, mitochondrial dysfunction, and oxidative stress (Carbone et al., 2022; Wang et al., 2022). Our findings suggest that the proteomic divergence between CRKP and CSKP is driven by these systemic insults rather than classical resistance mechanisms. PLXNB1, a member of the plexin axon guidance family, was significantly downregulated in the CRKP group. Beyond its role as a hub gene in Alzheimer’s disease and its association with amyloid plaques and tau pathology (Huang et al., 2024; Mathys et al., 2019; Yu et al., 2018), PLXNB1 is critically linked to endothelial integrity. It modulates BBB permeability, fibrin deposition, and endothelial tight junctions (Wu et al., 2020; Zhou et al., 2018). Its downregulation likely captures the exacerbated systemic microvascular and neurovascular injury characteristic of severe Gram-negative sepsis (Fazzari et al., 2007). We hypothesize that the persistent inflammatory state in CRKP infections drives this suppression, compromising vascular barrier function and contributing to sepsis-associated encephalopathy (Fazzari et al., 2007; Wang et al., 2015). Conversely, S100A1, a multifunctional Ca^2+^-binding protein (Kazakov et al., 2022), was significantly upregulated in CRKP patients. As a central regulator of cardiomyocyte signaling (Noll et al., 2024), its leakage into plasma—previously noted in myocardial infarction and acute ischemic stroke (AIS) (Heizmann, 2019; Hong et al., 2023)—stems from compromised membrane integrity induced by intense oxidative stress and mitochondrial dysfunction (Duarte-Costa et al., 2014). The specific elevation of S100A1 in CRKP likely reflects a more severe pathogen-specific myocardial insult compared to CSKP. The pronounced dysregulation of these markers highlights the intense immune–inflammatory response triggered by CRKP. This is consistent with evidence that CRKP drives a tenfold rise in IL-6 levels in mice compared to CSKP (Yang et al., 2024), suggesting that bacterial survival under antibiotic pressure accelerates disease progression. Both biomarkers are actively involved in these processes: PLXNB1 deficiency aggravates inflammation via impaired Treg function (Chapoval et al., 2024) and disrupts Rnd1-mediated production of pro-inflammatory cytokines like IL-6 and TNF-α (Kumar et al., 2022). Similarly, S100A1 acts as a DAMP, modulating NF-κB and cytokine expression (e.g., IL-6, IL-10) in both AIS and LPS-induced inflammation models (Hong et al., 2023; Bai et al., 2023; Yuan et al., 2025). These findings suggest that PLXNB1 and S100A1 are not merely markers of damage but active mediators of the systemic inflammatory injury that distinguishes CRKP from CSKP.

Despite these insights, our study has limitations. First, the sample size was relatively small, and the selection was limited to ICU patients with sepsis, which may restrict the generalizability of the findings. Second, as an exploratory investigation, these candidates were not validated by orthogonal methods like ELISA. However, our rigorous DIA-MS workflow (precursor-level FDR <1%) and high platform reproducibility support the technical validity of these candidates. Third, the absence of cohorts infected with other multidrug-resistant organisms (e.g., *A. baumannii*) makes it difficult to determine if this signature is CRKP-specific or a general response to severe Gram-negative sepsis. Nevertheless, their significant differential expression suggests value in stratifying resistance phenotypes within *Klebsiella* infections.

In conclusion, this study demonstrates the potential of plasma proteomics for early, non-culture-based CRKP diagnosis. Our two-protein model offers a preliminary theoretical basis for clinical decision-making in urgent ICU settings. Future studies involving diverse pathogen cohorts and targeted immunoassays are warranted to fully delineate the diagnostic specificity and clinical utility of PLXNB1 and S100A1.

## Data Availability

The raw data supporting the conclusions of this article will be made available by the authors, without undue reservation.

## References

[B1] Bai Y. Guo N. Xu Z. Chen Y. Zhang W. Chen Q. (2023). S100A1 expression is increased in spinal cord injury and promotes inflammation, oxidative stress and apoptosis of PC12 cells induced by LPS via ERK signaling. Mol. Med. Rep. 27 (2), 30. 10.3892/mmr.2022.12917 36524376 PMC9827259

[B2] Bassetti M. Peghin M. Pecori D. (2016). The management of multidrug-resistant enterobacteriaceae. Curr. Opin. Infect. Dis. 29 (6), 583–594. 10.1097/QCO.0000000000000314 27584587

[B3] Carbone F. Liberale L. Preda A. Schindler T. H. Montecucco F. (2022). Septic cardiomyopathy: from pathophysiology to the clinical setting. Cells 11 (18), 2833. 10.3390/cells11182833 36139408 PMC9496713

[B4] Chapoval S. P. Gao H. Fanaroff R. Keegan A. D. (2024). Plexin B1 controls Treg numbers, limits allergic airway inflammation, and regulates mucins. Front. Immunol. 14, 1297354. 10.3389/fimmu.2023.1297354 38259471 PMC10801081

[B5] Chen J. Yang Y. Yao H. Bu S. Li L. Wang F. (2022). Prediction of prognosis in adult patients with carbapenem-resistant Klebsiella pneumoniae infection. Front. Cell Infect. Microbiol. 11, 818308. 10.3389/fcimb.2021.818308 35087768 PMC8787092

[B6] Diekema D. J. Hsueh P. R. Mendes R. E. Pfaller M. A. Rolston K. V. Sader H. S. (2019). The microbiology of bloodstream infection: 20-Year trends from the SENTRY antimicrobial Surveillance program. Antimicrob. Agents Chemotherapy 63 (7). 10.1128/AAC.00355-19 31010862 PMC6591610

[B7] Duarte-Costa S. Castro-Ferreira R. Neves J. S. Leite-Moreira A. F. (2014). S100A1: a major player in cardiovascular performance. Physiol. Res. 63 (6), 669–681. 10.33549/physiolres.932712 25157660

[B8] Fazzari P. Penachioni J. Gianola S. Rossi F. Eickholt B. J. Maina F. (2007). Plexin-B1 plays a redundant role during mouse development and in tumour angiogenesis. BMC Dev. Biol. 7, 55. 10.1186/1471-213X-7-55 17519029 PMC1890291

[B9] Guan J. Ren Y. Dang X. Gui Q. Zhang W. Lu Z. (2025). Predictive model for carbapenem-resistant Klebsiella pneumoniae bloodstream infection based on a nomogram: a retrospective study. BMC Res. Notes 18 (1), 265. 10.1186/s13104-025-07325-w 40598640 PMC12220204

[B10] Hao L. Yang X. Chen H. Mo Z. Li Y. Wei S. (2022). Molecular characteristics and quantitative proteomic analysis of Klebsiella pneumoniae strains with Carbapenem and Colistin resistance. Antibiot. (Basel) 11 (10), 1341. 10.3390/antibiotics11101341 36289999 PMC9598126

[B11] Heizmann (2019). Heizmann CW. Ca-binding proteins of the EF-Hand superfamily: diagnostic and prognostic biomarkers and novel therapeutic targets. Methods Mol. Biol. 1929, 157–186. 10.1007/978-1-4939-9030-6_11 30710273

[B12] Hong G. Li T. Zhao H. Zeng Z. Zhai J. Li X. (2023). Diagnostic value and mechanism of plasma S100A1 protein in acute ischemic stroke: a prospective and observational study. PeerJ 11, e14440. 10.7717/peerj.14440 36643631 PMC9838205

[B13] Huang Y. Wang M. Ni H. Zhang J. Li A. Hu B. (2024). Regulation of cell distancing in peri-plaque glial nets by Plexin-B1 affects glial activation and amyloid compaction in Alzheimer's disease. Nat. Neurosci. 27 (8), 1489–1504. 10.1038/s41593-024-01664-w 38802590 PMC11346591

[B14] Kazakov A. S. Sofin A. D. Avkhacheva N. V. Deryusheva E. I. Rastrygina V. A. Permyakova M. E. (2022). Interferon-β activity is affected by S100B protein. Int. J. Mol. Sci. 23 (4), 1997. 10.3390/ijms23041997 35216109 PMC8877046

[B15] Kong H. Liu Y. Yang L. Chen Q. Li Y. Hu Z. (2025). Seven-year change of prevalence, clinical risk factors, and mortality of patients with carbapenem-resistant Klebsiella pneumoniae bloodstream infection in a Chinese teaching hospital: a case-case-control study. Front. Microbiol. 16, 1531984. 10.3389/fmicb.2025.1531984 40177489 PMC11962001

[B16] Kumar A. Mishra S. Kumar A. Raut A. A. Sato S. Takaoka A. (2022). Essential role of Rnd1 in innate immunity during viral and bacterial infections. Cell Death Dis. 13 (6), 520. 10.1038/s41419-022-04954-y 35654795 PMC9161769

[B17] Li Y. Kumar S. Zhang L. Wu H. (2022). Klebsiella pneumonia and its antibiotic resistance: a bibliometric analysis. BioMed Research International 2022, 1668789. 10.1155/2022/1668789 35707374 PMC9192197

[B18] Li N. Chen H. Li Y. Feng Y. Yu H. Liu S. (2024). Related factors of CRKP infection in neurosurgery and comparison of therapeutic effects of tigecycline *versus* polymyxin B for CRKP infection. Altern. Ther. Health Med. 30 (12), 112–116. 38466059

[B19] Liao Q. Feng Z. Chen X. (2023). Risk model and validation of carbapenem-resistant Klebsiella pneumoniae infection in patients with cerebrovascular disease in the ICU. Open Med. (Wars) 18 (1), 20230774. 10.1515/med-2023-0774 37663230 PMC10473460

[B20] Mangioni D. Chatenoud L. Colombo J. Palomba E. Guerrero F. A. Bolis M. (2023). Multidrug-Resistant bacterial colonization and infections in large retrospective cohort of mechanically ventilated COVID-19 patients. Emerg. Infect. Dis. 29 (8), 1598–1607. 10.3201/eid2908.230115 37486196 PMC10370845

[B21] Mathys H. Davila-Velderrain J. Peng Z. Gao F. Mohammadi S. Young J. Z. (2019). Single-cell transcriptomic analysis of Alzheimer’s disease. Nature 570, 332–337. 10.1038/s41586-019-1195-2 31042697 PMC6865822

[B22] Noll D. Kehr D. Most P. Ritterhoff J. (2024). S100A1: a promising therapeutic target for heart failure. Expert Opin. Ther. Targets 28 (4), 233–236. 10.1080/14728222.2024.2345746 38641766

[B23] Sekino N. Selim M. Shehadah A. (2022). Sepsis-associated brain injury: underlying mechanisms and potential therapeutic strategies for acute and long-term cognitive impairments. J. Neuroinflammation 19 (1), 101. 10.1186/s12974-022-02464-4 35488237 PMC9051822

[B24] Shu T. Ning W. Wu D. Xu J. Han Q. Huang M. (2020). Plasma proteomics identify biomarkers and pathogenesis of COVID-19. Immunity 53 (5), 1108–1122.e5. 10.1016/j.immuni.2020.10.008 33128875 PMC7574896

[B25] The predictive value of inflammatory. The predictive value of inflammatory biomarkers in the detection of multiple sclerosis attacks. (2023). Emerg. Care J., 19(2). 10.4081/ecj.2023.11314

[B26] Vural N. Duyan M. Saridas A. Ertas E. (2024). Evaluation of inflammatory biomarkers affecting mortality in acute cholecystitis in the emergency department. Bratisl. Lek. Listy 125 (6), 365–370. 10.4149/BLL_2024_55 38757593

[B27] Wan S. Zhou A. Chen R. Fang S. Lu J. Lv N. (2024). Metagenomics next-generation sequencing (mNGS) reveals emerging infection induced by Klebsiella pneumoniaeniae. Int. J. Antimicrob. Agents 63 (2), 107056. 10.1016/j.ijantimicag.2023.107056 38081548

[B28] Wang H. Hong L. J. Huang J. Y. Jiang Q. Tao R. R. Tan C. (2015). P2RX7 sensitizes Mac-1/ICAM-1-dependent leukocyte-endothelial adhesion and promotes neurovascular injury during septic encephalopathy. Cell Res. 25 (6), 674–690. 10.1038/cr.2015.61 25998681 PMC4456628

[B29] Wang R. Xu Y. Fang Y. Wang C. Xue Y. Wang F. (2022). Pathogenetic mechanisms of septic cardiomyopathy. J. Cell Physiol. 237 (1), 49–58. 10.1002/jcp.30527 34278573

[B30] Wang G. Cao L. Lian L. Wang Y. Lian J. Liu Z. (2025). Machine learning and DIA proteomics reveal new insights into carbapenem resistance mechanisms in Klebsiella pneumoniae. J. Proteome Res. 24 (8), 4002–4014. 10.1021/acs.jproteome.5c00142 40622342

[B31] Wei D. Melgarejo J. D. Van Aelst L. Vanassche T. Verhamme P. Janssens S. (2023). Prediction of coronary artery disease using urinary proteomics. Eur. J. Prev. Cardiol. 30 (14), 1537–1546. 10.1093/eurjpc/zwad087 36943304

[B32] Wu J. H. Li Y. N. Chen A. Q. Hong C. D. Zhang C. L. Wang H. L. (2020). Inhibition of Sema4D/PlexinB1 signaling alleviates vascular dysfunction in diabetic retinopathy. EMBO Mol. Med. 12 (2), e10154. 10.15252/emmm.201810154 31943789 PMC7005627

[B33] Wu C. Zheng L. Yao J. (2022). Analysis of risk factors and mortality of patients with carbapenem-resistant Klebsiella pneumoniae infection. Infect. Drug Resist 15, 2383–2391. 10.2147/IDR.S362723 35535031 PMC9078358

[B34] Xu L. Sun X. Ma X. (2017). Systematic review and meta-analysis of mortality of patients infected with carbapenem-resistant Klebsiella pneumoniae. Ann. Clinical Microbiology Antimicrobials 16 (1), 18. 10.1186/s12941-017-0191-3 28356109 PMC5371217

[B35] Yang C. Xu Q. Xie M. Tang Y. Hu Q. Heng H. (2024). Enhancing resistance, but not virulence attributed to the high mortality caused by carbapenem-resistant Klebsiella pneumoniae. Microbiol. Res. 285, 127769. 10.1016/j.micres.2024.127769 38797112

[B36] Yu L. Petyuk V. A. Gaiteri C. Mostafavi S. Young-Pearse T. Shah R. C. (2018). Targeted brain proteomics uncover multiple pathways to Alzheimer’s dementia. Ann. Neurol. 84, 78–88. 10.1002/ana.25266 29908079 PMC6119500

[B37] Yuan S. Huang Y. Wang Q. Shi Y. Song X. R. Zhao Z. (2025). Roles of S100A1 and S100A10 from hybrid grouper (epinephelus lanceolatus♂ × epinephelus fuscoguttatus♀) in immune response to Vibrio infection. Fish. Shellfish Immunol. 157, 110070. 10.1016/j.fsi.2024.110070 39631555

[B38] Zhang J. Wei Z. Qi X. Hou X. Liu D. He J. (2023). Integrative proteomics, phosphoproteomics and acetylation proteomics analyses of acute pancreatitis in rats. Int. Journal Medical Sciences 20 (7), 888–900. 10.7150/ijms.81658 37324185 PMC10266050

[B39] Zhou Y. F. Li Y. N. Jin H. J. Wu J. H. He Q. W. Wang X. X. (2018). Sema4D/PlexinB1 inhibition ameliorates blood-brain barrier damage and improves outcome after stroke in rats. FASEB J. 32 (4), 2181–2196. 10.1096/fj.201700786RR 29242274

